# Digital subtraction pancreatography enhances ductal visualization in high grade pancreatic intraepithelial neoplasia

**DOI:** 10.1055/a-2718-4846

**Published:** 2025-10-29

**Authors:** Akihiro Maruyama, Kohei Takano, Junya Yamada, Hiroki Kato, Sakurako Isobe, Makoto Kobayashi

**Affiliations:** 1 37036 Department of Gastroenterology, Yokkaichi Municipal Hospital, Yokkaichi, Japan

Digital subtraction imaging (DSI) is a radiological technique used to enhance the visualization of anatomical structures by subtracting pre-contrast images from post-contrast ones, thereby effectively eliminating overlapping background signals 1 . While widely utilized in angiography and interventional radiology, recent reports have suggested its utility in gastrointestinal imaging as well 2 3 .

We present a case of a 69-year-old man referred for further evaluation due to worsening glycemic control and mildly elevated serum CA19–9. Cross-sectional imaging revealed stenosis of the main pancreatic duct (MPD) in the pancreatic body, along with upstream ductal dilatation, but failed to identify any obvious mass. To further evaluate the MPD, endoscopic retrograde pancreatography was performed under conscious sedation with intravenous midazolam. The procedure was conducted using TJF-260V (Olympus, Tokyo, Japan) and Tandem XL Triple Lumen ERCP Cannula (Boston Scientific, Marlborough, Massachusetts, USA), with amidotrizoic acid as the contrast medium. Contrast injection was limited to the point at which branch pancreatic ducts became visible to minimize intraductal pressure. Both conventional fluoroscopy and DSI were utilized ( Video 1 ). The DSI sequence was acquired using Ultimax-i DREX-U180 (Canon Medical Systems, Tokyo, Japan) and clearly delineated the branch pancreatic ducts in the pancreatic body by removing overlying vertebral shadows ( Fig. 1 ). The additional radiation dose from DSI was minimal (2.1 mGy), and no post-procedural adverse events occurred. Although DSI may be affected by respiratory motion artifacts 4 , the acquisition of multiple sequential frames allows for the selection of optimal images synchronized with stable respiratory phases.

Digital subtraction pancreatography during endoscopic retrograde pancreatography clarified branch duct visualization by removing vertebral overlap, enabling clearer evaluation of subtle pancreatic duct abnormalities.Video 1

**Fig. 1:**
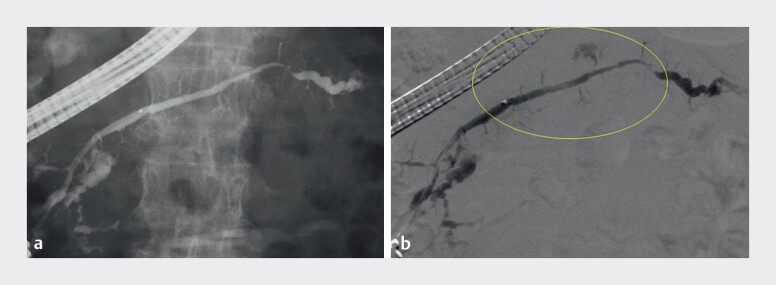
**a** Conventional fluoroscopic image during endoscopic retrograde pancreatography shows the main pancreatic duct with limited visibility of the surrounding structures. **b** Digital subtraction pancreatography clearly delineates the branch pancreatic ducts in the body region. The yellow circle highlights the area where overlapping vertebral shadows have been eliminated, allowing improved visualization of the pancreatic ductal anatomy.

Serial pancreatic juice aspiration cytologic examination raised suspicion of malignancy. The patient subsequently underwent distal pancreatectomy. Histological examination confirmed the diagnosis of high grade pancreatic intraepithelial neoplasia ( Fig. 2 ).

**Fig. 2:**
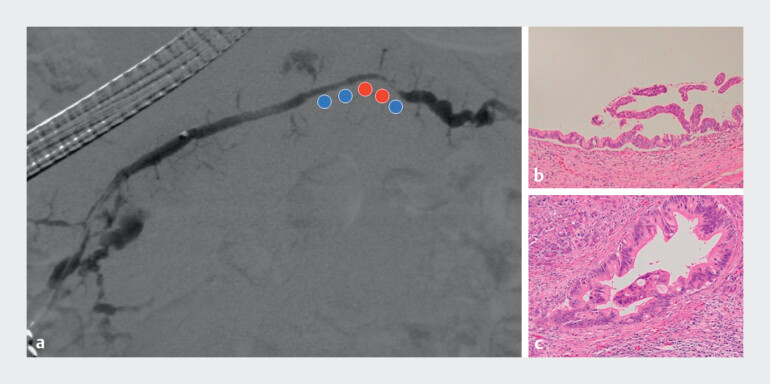
**a** Digital subtraction pancreatography with histopathological correlation. Blue circles indicate regions corresponding to low grade pancreatic intraepithelial neoplasia (PanIN), while red circles indicate regions corresponding to high grade PanIN. **b** Hematoxylin and eosin (H&E) staining of the surgical specimen from the region indicated by the blue circles reveals low grade PanIN with mild architectural and cytological atypia (×20). **c** H&E staining of the region indicated by the red circles demonstrates high grade PanIN with prominent nuclear atypia and architectural complexity (×20).

This case illustrates the potential of digital subtraction pancreatography to improve visualization of subtle ductal abnormalities in the pancreatic body, particularly when conventional imaging is inconclusive.

Endoscopy_UCTN_Code_TTT_1AR_2AB

## Notes

Endoscopy E-Videos https://eref.thieme.de/e-videos
*E-Videos* is an open access online section of the journal *Endoscopy* , reporting on interesting cases and new techniques in gastroenterological endoscopy. All papers include a high-quality video and are published with a Creative Commons CC-BY license. Endoscopy E-Videos qualify for HINARI discounts and waivers and eligibility is automatically checked during the submission process. We grant 100% waivers to articles whose corresponding authors are based in Group A countries and 50% waivers to those who are based in Group B countries as classified by Research4Life (see: https://www.research4life.org/access/eligibility/ ). This section has its own submission website at https://mc.manuscriptcentral.com/e-videos .
